# Hypoxia-induced metastasis of human melanoma cells: involvement of vascular endothelial growth factor-mediated angiogenesis

**DOI:** 10.1038/sj.bjc.6690586

**Published:** 1999-08

**Authors:** E K Rofstad, T Danielsen

**Affiliations:** Department of Biophysics, Institute for Cancer Research, Oslo, The Norwegian Radium Hospital, Montebello, 0310, Norway

**Keywords:** angiogenesis, hypoxia, melanoma, metastasis, VEGF

## Abstract

Tumour cells exposed to hypoxia have been shown to up-regulate the expression of vascular endothelial growth factor (VEGF). The purpose of the present work was to investigate whether hypoxia-induced VEGF up-regulation can result in increased metastatic efficiency of human melanoma cells. Two melanoma lines, one showing high (A-07) and the other showing low (D-12) VEGF secretion under aerobic conditions, were included in the study. Cell cultures were exposed to hypoxia (oxygen concentrations < 10 ppm) in vitro and metastatic efficiency, i.e. lung colonization efficiency, as well as transplantability and angiogenic potential were assessed in BALB/c-*nu/nu* mice. Both cell lines showed significantly increased VEGF secretion under hypoxic conditions as measured by enzyme-linked immunosorbent assay. The D-12 cells showed increased metastatic efficiency, transplantability and angiogenic potential following exposure to hypoxia. The metastatic efficiency increased with the duration of the hypoxia treatment and decreased with the time after reoxygenation. The A-07 cells on the other hand showed unchanged metastatic efficiency, transplantability and angiogenic potential following exposure to hypoxia. Both cell lines showed significantly decreased metastatic efficiency and angiogenic potential in mice treated with neutralizing antibody against VEGF. These results suggest that (a) VEGF is a limiting factor for the rate of angiogenesis in low but not in high VEGF-expressing melanomas under normoxic conditions and (b) transient hypoxia might promote the development of metastases in low VEGF-expressing melanomas by upregulating the expression of VEGF and hence enhancing the angiogenic potential of the tumour cells. © 1999 Cancer Research Campaign


*British Journal of Cancer* (1999) **80**(11), 1697–1707
© 1999 Cancer Research Campaign
Article no. bjoc.1999.0586

### Hypoxia-induced metastasis of human melanoma cells: involvement of vascular endothelial growth factor-mediated angiogenesis

**EK Rofstad and T Danielsen**

Department of Biophysics, Institute for Cancer Research, The Norwegian Radium Hospital, Montebello, 0310 Oslo, Norway

**Summary** Tumour cells exposed to hypoxia have been shown to up-regulate the expression of vascular endothelial growth factor (VEGF). The purpose of the present work was to investigate whether hypoxia-induced VEGF up-regulation can result in increased metastatic efficiency of human melanoma cells. Two melanoma lines, one showing high (A-07) and the other showing low (D-12) VEGF secretion under aerobic conditions, were included in the study. Cell cultures were exposed to hypoxia (oxygen concentrations < 10 ppm) in vitro and metastatic efficiency, i.e. lung colonization efficiency, as well as transplantability and angiogenic potential were assessed in BALB/c-*nu/nu* mice. Both cell lines showed significantly increased VEGF secretion under hypoxic conditions as measured by enzyme-linked immunosorbent assay. The D-12 cells showed increased metastatic efficiency, transplantability and angiogenic potential following exposure to hypoxia. The metastatic efficiency increased with the duration of the hypoxia treatment and decreased with the time after reoxygenation. The A-07 cells on the other hand showed unchanged metastatic efficiency, transplantability and angiogenic potential following exposure to hypoxia. Both cell lines showed significantly decreased metastatic efficiency and angiogenic potential in mice treated with neutralizing antibody against VEGF. These results suggest that (a) VEGF is a limiting factor for the rate of angiogenesis in low but not in high VEGF-expressing melanomas under normoxic conditions and (b) transient hypoxia might promote the development of metastases in low VEGF-expressing melanomas by upregulating the expression of VEGF and hence enhancing the angiogenic potential of the tumour cells.

**Keywords:** angiogenesis; hypoxia; melanoma; metastasis; VEGF

Tumour tissue develops an inadequate vasculature and an abnormal physiological microenvironment during growth (Vaupel et al, 1989). Angiogenesis, i.e. the process of generation and development of capillary blood vessels, is necessary for a tumour to grow beyond a certain size given by the diffusion distances of oxygen and other nutrients (Folkman, 1990). The rate of angiogenesis is usually not sufficiently high to keep up with the rate of proliferation of the malignant tumour cells (Denekamp and Hobson, 1982). The capillary network of tumours therefore shows characteristic architectural and functional abnormalities, which result in insufficient oxygen supply and the development of regions of hypoxic tissue (Coleman, 1988; Vaupel et al, 1989). Two types of hypoxia have been recognized in tumours: chronic hypoxia, arising from limitations in oxygen diffusion, and acute hypoxia, resulting from transient stoppages in microregional blood flow (Horsman, 1995). Chronically hypoxic cells usually lie remotely from capillaries, whereas acutely hypoxic cells can be found adjacent to capillaries (Coleman, 1988; Horsman, 1995). Metastasis, i.e. the spread of malignant tumour cells from the primary neoplasm to distant sites, is influenced by the angiogenic potential and the oxygenation status of the tumour cells (Hill, 1990; Weidner, 1993).

*Received 15 October 1998*
*Revised 5 January 1999*
*Accepted 28 January 1999*

*Correspondence to:* EK Rofstad

The relationship between tumour metastasis and angiogenesis has been demonstrated in a series of clinical studies involving a wide range of histological types of tumours (Weidner et al, 1991; Macchiarini et al, 1992; Gasparini et al, 1993; Bochner et al, 1995). These studies showed that the probability of metastasis is positively correlated to the vascular density of the primary tumour and led to the suggestion that vascular density might be an independent prognostic indicator in malignant diseases (Weidner, 1993). The metastatic propensity of a tumour might be influenced by the angiogenic potential of the tumour cells by two independent mechanisms: high vascular density in the primary tumour might enhance the opportunity of the tumour cells to gain access to the blood stream and elevated capacity to induce neovascularization might increase the probability of tumour cells trapped in distant organ capillary beds to give rise to macroscopic tumour growth (Fidler and Ellis, 1994).

Significant correlations between tumour metastasis and oxygenation or between tumour metastasis and lactate concentration have been demonstrated in clinical studies involving soft tissue sarcoma (Brizel et al, 1996), cervix carcinoma (Schwickert et al, 1995; Höckel et al, 1996; Sundfør et al, 1998) and carcinoma of the head and neck (Walenta et al, 1997). High lactate concentration is indicative of extensive anaerobic metabolism and hence poor oxygenation in tumour tissue (Vaupel et al, 1989). These studies are apparently inconsistent with those showing correlations between tumour metastasis and angiogenesis as primary tumours with low oxygen tensions or high lactate concentrations were found to metastasize more frequently than primary tumours with high oxygen tensions or low lactate concentrations (Schwickert et al, 1995; Brizel et al, 1996; Höckel et al, 1996; Walenta et al, 1997; Sundfør et al, 1998). However, they are consistent with a study of experimental murine tumours which showed that tumour cells exposed to transient hypoxia in vitro are more metastatic than aerobic control cells when injected intravenously (i.v.) in syngeneic mice (Young et al, 1988).

Tumour cells exposed to hypoxia in vitro or in vivo show increased expression of several selected genes, including the gene encoding vascular endothelial growth factor (VEGF) (Dachs and Stratford, 1996). VEGF is a potent angiogenic factor existing in four different isoforms ($VEGF_{121,165,189,206}$) arising from alternative *m*-RNA splicing (Zagzag, 1995). The expression of VEGF is correlated to vascular density in several histological types of human tumours (Guidi et al, 1995; Toi et al, 1995; Takahashi et al, 1995; Mattern et al, 1996). Xenografted tumours established from cell lines transfected with VEGF show increased vascular density, volumetric growth rate and metastatic frequency relative to wild-type control tumours (Zhang et al, 1995; Claffey et al, 1996; Pötgens et al, 1996), whereas xenografted tumours initiated from cell lines transfected with antisense-VEGF *c*-DNA show reduced vascular density and growth (Saleh et al, 1996). The neovascularization of experimental tumours is inhibited by treatment with monoclonal antibodies against VEGF (Kim et al, 1993; Asano et al, 1995; Melnyk et al, 1996).

The possibility thus exists that hypoxic tumour cells after dissemination have increased capacity to induce neovascularization in secondary organ sites and hence are more metastatic than their normoxic counterparts, owing to increased VEGF expression induced by the hypoxic microenvironment. The purpose of the work reported here was to test this hypothesis. Cells from two human melanoma lines differing significantly in VEGF synthesis and secretion were exposed to hypoxia in vitro. Metastatic efficiency, i.e. lung colonization efficiency, was assessed in athymic nude mice after i.v. administration of tumour cells. Transplantability and angiogenic potential were also determined in vivo, using intradermal (i.d.) assays in athymic nude mice. Hypoxia-induced VEGF up-regulation was studied in vitro using Western blot analysis and ELISA.

### MATERIALS AND METHODS

### Mice

Adult (8–10 weeks old) female BALB/c-*nu*/*nu* mice were used as host animals to assess tumour cell metastatic efficiency, transplantability and angiogenic potential. The mice were bred at our institute and maintained under specific pathogen-free conditions at constant temperature (24–26°C) and humidity (30–50%). Sterilized food and tap water were given ad libitum.

### Cell lines

Two human melanoma cell lines (A-07 and D-12) giving rise to pulmonary metastases in athymic nude mice were included in the study (Rofstad, 1994). The cell lines were maintained in monolayer culture in RPMI-1640 medium (25 mM HEPES and L-glutamine) supplemented with 13% fetal calf serum, 250 mg $l^{-1}$ penicillin and 50 mg $l^{-1}$ streptomycin. The cultures were incubated at 37°C in a humidified atmosphere of 5% carbon dioxide in air and subcultured twice a week by trypsinization (treatment with 0.05% trypsin–0.02% EDTA solution at 37°C for 2 min). The cell lines were verified to be free from *Mycoplasma* contamination by using the Hoechst fluorescence and the mycotrin methods.

### Hypoxia exposure

Monolayer cell cultures growing in glass dishes were exposed to hypoxia for 0–24 h by using the steel-chamber method (Sanna and Rofstad, 1994). The cultures were incubated at 37°C in a humidified atmosphere of 5% carbon dioxide in air for 24 h before the hypoxia treatment. The culture medium was removed and replaced by fresh medium immediately before the cells were exposed to hypoxia. The medium used during the hypoxia treatment was supplemented with 2.2 g $l^{-1}$ sodium bicarbonate. The pH of the medium was 7.4 ± 0.1. The glass dishes were kept in air-tight steel chambers during the hypoxia treatment. The medium layer covering the cells was approximately 2 mm in thickness. The steel chambers were flushed with a humidified, highly purified gas mixture consisting of 95% nitrogen and 5% carbon dioxide at a flow rate of 5 l $min^{-1}$. Measurements showed that the concentration of oxygen in the medium was less than 10 ppm after 30 min of flushing. Control cultures were flushed with humidified 5% carbon dioxide in air. The cells were reoxygenated by opening the steel-chambers. The cultures were kept in fresh medium in humidified 5% carbon dioxide in air for reoxygenation times of 0–24 h before the cells were detached from the glass dishes by trypsinization and washed in $Ca^{2+}$- and $Mg^{2+}$-free Hanks' balanced salt solution (HBSS). Cell aggregates were not seen in any of the suspensions.

### Cell viability assay

The viability of cells from control and hypoxia-treated cultures was assessed by measuring plating efficiency (PE) in vitro (Rofstad, 1992). Aliquots of $2.0 \times 10^2$ cells suspended in 1 ml of fresh medium were plated in 25-$cm^2$ culture flasks. The flasks contained approximately $1.0 \times 10^5$ lethally irradiated (30 Gy) feeder cells in 4 ml of medium, plated 24 h earlier. The cells were incubated at 37°C in a humidified atmosphere of 5% carbon dioxide in air for 10 days. The medium was removed and replaced by fresh medium on day 7. The cells were fixed in 100% ethanol and stained with methylene blue. Colonies containing more than 50 cells were counted using a stereomicroscope.

### Metastasis assay

Metastatic efficiency was assessed by using a lung colonization assay (Rofstad, 1995). Aliquots of $2.0 \times 10^6$–$3.0 \times 10^6$ A-07 cells or $5.0 \times 10^5$ D-12 cells suspended in 0.2 ml of HBSS were inoculated into the lateral tail vein of mice by using a tuberculin syringe with a 26-gauge needle. The mice were killed and autopsied 5 weeks after the inoculation. The lungs were removed, rinsed in HBSS and fixed in Bouin's solution for 24 h to facilitate the scoring of colonies. The number of surface colonies was recorded using a stereomicroscope. Metastatic efficiency in terms of number of lung colonies per $1.0 \times 10^6$ viable A-07 cells or per $1.0 \times 10^5$ viable D-12 cells was determined by correcting the number of colonies for the number of cells per inoculum and the PE measured in vitro, as recommended by Young et al (1988).

© 1999 Cancer Research Campaign

### Transplantation assay

Transplantability was assessed using an intradermal transplantation assay (Rofstad, 1995). A 100-μl Hamilton syringe was used to inoculate aliquots of 10 μl of cell suspension into the flank of mice. The cells were suspended in HBSS, and the number of cells per inoculum was varied within the ranges $2.0 \times 10^3$–$2.0 \times 10^5$ (A-07) and $2.0 \times 10^2$–$5.0 \times 10^4$ (D-12). The inoculation point lay above the subcutaneous muscle tissue in the deeper part of the dermis. The mice were examined twice weekly for up to 120 days after the inoculation. A transplantation was scored as positive when the tumour diameters reached 10–12 mm, and then the host was killed. All positive transplantations were scored before day 90 after the inoculation. The fraction of positive transplantations was plotted vs the number of cells per inoculum, and probit regression lines were fitted to the data.

### Angiogenesis assay

Angiogenic potential was assessed using an i.d. angiogenesis assay (Kreisle and Ershler, 1988). Aliquots of 10 μl of cell suspension were inoculated i.d. in mice, using the same procedure as used in the transplantability studies described above. The number of cells per inoculum was varied within the range $2.5 \times 10^4$–$1.3 \times 10^6$. The mice were killed on day 5 (A-07) or day 7 (D-12) after the inoculation. Small vascularized tumours had developed in the inoculation sites at that time. The skin around the inoculation sites was removed and the tumours were located with a dissecting microscope. Angiogenesis was quantified by counting the capillaries in the dermis oriented towards the tumours (Rofstad, 1994). The number of capillaries was corrected for the background, determined after the injection of 10 μl HBSS. The tumours were dissected free from the skin and weighed after the tumour-oriented capillaries had been scored. The number of capillaries was plotted versus the number of cells per inoculum, and linear regression lines were fitted to the data.

### Treatment with anti-VEGF neutralizing antibody

The specific role of VEGF in metastasis and angiogenesis was investigated by assessing metastatic efficiency and angiogenic potential in mice treated with the anti-human VEGF neutralizing monoclonal antibody MAB293 (R&D Systems, Inc., Minneapolis, MN, USA). This antibody is produced from a murine hybridoma elicited from a mouse immunized with purified insect cell line *Sf* 21-derived recombinant human VEGF. The antibody does not cross-react with human platelet derived growth factor. Two doses of MAB293 of 25 μg in 0.2 ml of phosphate-buffered saline (PBS) were administered to the mice by intraperitoneal (i.p.) injection. The first dose was given 1 h before and the second dose 23 h after tumour cells were inoculated either into the lateral tail vein for formation of lung colonies or i.d. for evocation of angiogenesis and tumour formation.

### Western blot analysis of intracellular VEGF

Cells were washed in PBS and boiled in Laemmli sample buffer for 5 min (Laemmli, 1970). Proteins were separated by electrophoresis in 13% sodium dodecyl sulphate-polyacrylamide gels and blotted to polyvinylidene difluoride transfer membranes (Towbin et al, 1979). Membranes were incubated with the A-20 anti-VEGF rabbit polyclonal antibody (Santa Cruz Biotechnology, Inc., Santa Cruz, CA, USA) for 60 min. Bound antibody was detected using a biotin–streptavidin alkaline phosphatase staining procedure.

### ELISA analysis of VEGF secretion

VEGF concentration in culture medium was measured using a commercially available human $VEGF_{165}$ enzyme-linked immunosorbent assay (ELISA) kit (R&D Systems, Inc., Minneapolis, MN, USA) according to manufacturer's instructions. Medium samples were collected from cell cultures immediately before and after a 24-h hypoxia treatment, centrifuged to remove particulates, and assayed in duplicate. Absorbances were read at 450 nm. Readings at 570 nm were subtracted from the readings at 450 nm to correct for optical imperfections in the plates. Rate of VEGF secretion ($R_{sec}$) was calculated as

$$R_{sec} = \frac{V\Delta C}{N_i \Delta t} \times \frac{1n(N_f / N_i)}{(N_f / N_i - 1)}$$

where $\Delta C$ is the increase in VEGF concentration during the time interval $\Delta t$ (24 h), $N_i$ and $N_f$ are the initial and final cell numbers and $V$ is the volume of medium. The second factor of this product is based on the assumption that the cell number increased exponentially with time during $\Delta t$, an assumption which was verified to be fulfilled for aerobic control cultures. There was no cell proliferation under hypoxic conditions, i.e. the second factor of the product was ~ 1 for hypoxic cultures. Replicate cultures were used to determine $N_i$. Cell numbers were counted using a haemocytometer. The fraction of Trypan-blue-excluding cells after treatment was > 95% and similar for control and hypoxia-treated cultures.

### Statistical analysis

Results are presented as arithmetic mean ± s.e.m. Statistical comparisons of mean values were performed under conditions of normality and equal variance by using the Student's *t*-test for single comparisons and one-way analysis of variance (ANOVA) and the Student–Newman–Keuls test for multiple comparisons. All *P*-values were determined from two-sided tests. Probability values of $P < 0.05$ were considered significant. The statistical analysis was performed using SigmaStat statistical software (Jandel Scientific GmbH, Erkrath, Germany).

### RESULTS

Exposure to hypoxia resulted in a transient increase in the metastatic efficiency of the D-12 cells (Figure 1). The metastatic efficiency was highest immediately after the reoxygenation. It increased with the duration of the hypoxia treatment and decreased with the reoxygenation time. A hypoxia treatment of 4 h was sufficient to increase the metastatic efficiency significantly. Thus, the metastatic efficiency of cell cultures exposed to hypoxia for 4 h was significantly higher than that of aerobic control cultures, whether the hypoxia-treated cultures were assayed immediately (< 1 h) after ($P < 0.01$) or 4 h after ($P < 0.05$) the reoxygenation. The maximum increase in metastatic efficiency was most likely reached after a hypoxia treatment of 16–24 h. Cell cultures exposed to hypoxia for 24 h and assayed immediately after or

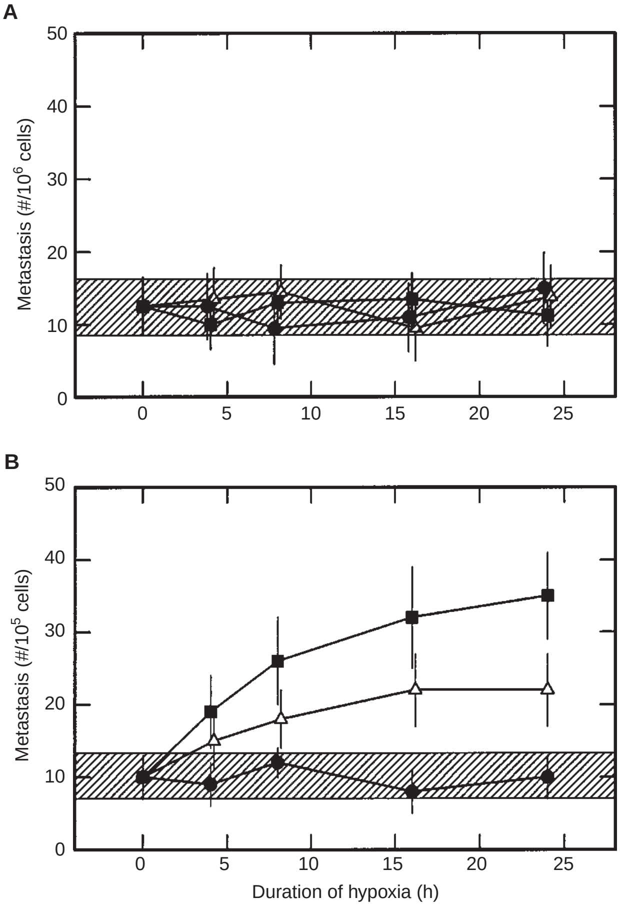

**Figure 1** A-07 (**A**) and D-12 (**B**) cells were exposed to hypoxia in vitro, reoxygenated and inoculated intravenously in female BALB/c-*nu/nu* mice for formation of lung colonies. The lung colonies were scored 5 weeks after the inoculation. The plots show number of lung colonies per $1.0 \times 10^6$ viable A-07 cells or per $1.0 \times 10^5$ viable D-12 cells vs time under hypoxic conditions. The cells were inoculated immediately (< 1 h) after the reoxygenation (■), 4 h after the reoxygenation (Δ) or 24 h after the reoxygenation (●). Mean values (points) and s.e.m. (bars) of 6–10 experiments, each involving 10 mice. The hatched horisontal bars refer to aerobic control cultures (mean values ± s.e.m.)

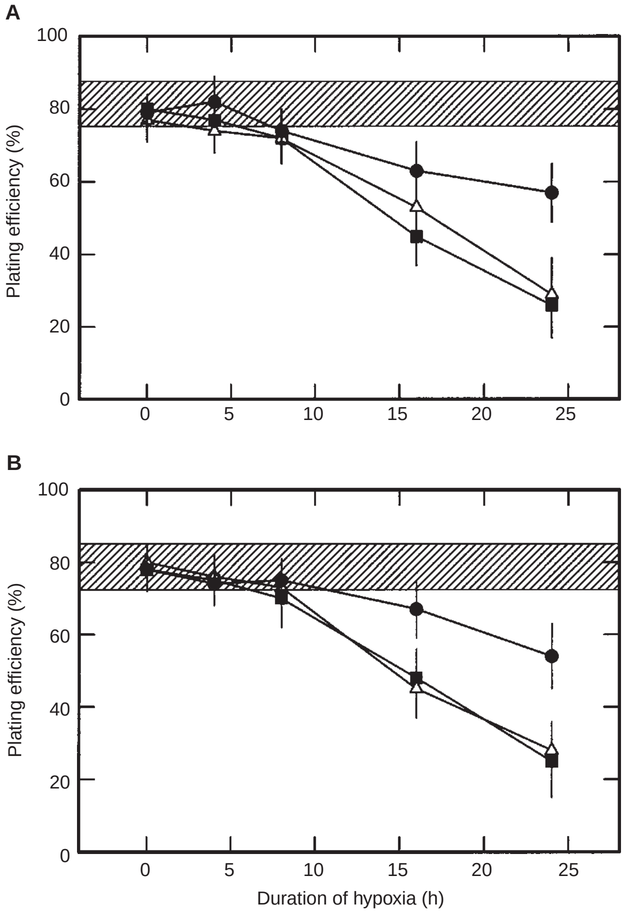

**Figure 2** A-07 (**A**) and D-12 (**B**) cells were exposed to hypoxia in vitro, reoxygenated and plated in tissue culture flasks for colony formation. The colonies were scored 10 days after the plating. The plots show PE vs time under hypoxic conditions. The cells were plated immediately (< 1 h) after the reoxygenation (■), 4 h after the reoxygenation (Δ) or 24 h after the reoxygenation (●). Mean values (points) and s.e.m. (bars) of 6–10 experiments. The hatched horisontal bars refer to aerobic control cultures (mean values ± s.e.m.)

4 h after the reoxygenation showed a metastatic efficiency which was higher than that of aerobic control cultures by factors of 3–4 ($P < 0.005$) and 2–3 ($P < 0.01$) respectively. A reoxygenation time of 24 h was sufficient to eliminate the hypoxia-induced increase in metastatic efficiency. Thus, the metastatic efficiency of cell cultures exposed to hypoxia for 24 h and assayed 24 h after the reoxygenation was not significantly different from that of aerobic control cultures ($P > 0.05$). In contrast, exposure to hypoxia did not change the metastatic efficiency of the A-07 cells (Figure 1); cell cultures exposed to hypoxia for 4, 8, 16 or 24 h and assayed immediately after, 4 h after or 24 h after the reoxygenation showed a metastatic efficiency which was not significantly different from that of aerobic control cultures ($P > 0.05$). The metastasis data presented in Figure 1 were calculated by taking into consideration the PE in vitro of the cell cultures (Table 1). The PE values of cell cultures treated equally were not significantly different for A-07 and D-12 cells ($P > 0.05$; Figure 2).

The transplantability of the D-12 cells also increased transiently by exposure to hypoxia, independent of the number of cells per inoculum (Figure 3). It was highest immediately after the reoxygenation and decreased with the reoxygenation time, similar to the metastatic efficiency. Thus, the transplantability of cell cultures exposed to hypoxia for 24 h was significantly higher than that of aerobic control cultures when the hypoxia-treated cultures were assayed immediately after ($P < 0.005$) or 4 h after the reoxygenation ($P < 0.01$). Moreover, the cell cultures assayed immediately after the reoxygenation showed a significantly higher transplantability than the cell cultures assayed 4 h after the reoxygenation ($P < 0.01$). A reoxygenation time of 24 h was sufficient to eliminate the hypoxia-induced increase in transplantability; cell cultures assayed 24 h after the reoxygenation showed a transplantability which was not significantly different from that of aerobic control cultures ($P > 0.05$; data not shown). In contrast, exposure to hypoxia did not change the transplantability of the A-07 cells (Figure 3); the transplantability of cell cultures exposed to hypoxia for 24 h and assayed immediately after or 4 h after the reoxygenation was not significantly different from that of aerobic control cultures ($P > 0.05$).

*© 1999 Cancer Research Campaign*

**Table 1** Data regarding the assessment of metastatic efficiency of A-07 and D-12 cells

| Time between reoxygenation and cell inoculation (h) | Duration of hypoxia treatment (h) | | | | | | | | | |
|---|---|---|---|---|---|---|---|---|---|---|
| | A-07 | | | | | D-12 | | | | |
| | 0 | 4 | 8 | 16 | 24 | 0 | 4 | 8 | 16 | 24 |
| Number of cells per inoculum | | | | | | | | | | |
| 0 | $2.0 \times 10^6$ | $2.0 \times 10^6$ | $2.0 \times 10^6$ | $3.0 \times 10^6$ | $3.0 \times 10^6$ | $5.0 \times 10^5$ | $5.0 \times 10^5$ | $5.0 \times 10^5$ | $5.0 \times 10^5$ | $5.0 \times 10^5$ |
| 4 | $2.0 \times 10^6$ | $2.0 \times 10^6$ | $2.0 \times 10^6$ | $3.0 \times 10^6$ | $3.0 \times 10^6$ | $5.0 \times 10^5$ | $5.0 \times 10^5$ | $5.0 \times 10^5$ | $5.0 \times 10^5$ | $5.0 \times 10^5$ |
| 24 | $2.0 \times 10^6$ | $2.0 \times 10^6$ | $2.0 \times 10^6$ | $3.0 \times 10^6$ | $3.0 \times 10^6$ | $5.0 \times 10^5$ | $5.0 \times 10^5$ | $5.0 \times 10^5$ | $5.0 \times 10^5$ | $5.0 \times 10^5$ |
| Number of lung colonies | | | | | | | | | | |
| 0 | 19 ± 6[a] | 17 ± 4 | 16 ± 4 | 20 ± 4 | 9 ± 4 | 41 ± 8 | 69 ± 13 | 91 ± 15 | 79 ± 12 | 44 ± 5 |
| 4 | 21 ± 4 | 22 ± 5 | 21 ± 4 | 14 ± 7 | 12 ± 5 | 40 ± 6 | 59 ± 10 | 64 ± 11 | 49 ± 8 | 29 ± 5 |
| 24 | 17 ± 5 | 21 ± 5 | 17 ± 6 | 19 ± 9 | 25 ± 7 | 38 ± 8 | 31 ± 8 | 48 ± 5 | 26 ± 7 | 28 ± 6 |
| Plating efficiency in vitro (%) | | | | | | | | | | |
| 0 | 80 ± 4[b] | 77 ± 6 | 72 ± 7 | 45 ± 8 | 26 ± 9 | 78 ± 6 | 75 ± 7 | 70 ± 8 | 48 ± 8 | 25 ± 10 |
| 4 | 77 ± 6 | 74 ± 6 | 72 ± 6 | 53 ± 8 | 29 ± 10 | 80 ± 5 | 76 ± 6 | 73 ± 6 | 45 ± 8 | 28 ± 8 |
| 24 | 79 ± 5 | 82 ± 7 | 74 ± 6 | 63 ± 8 | 57 ± 8 | 78 ± 5 | 74 ± 6 | 75 ± 6 | 67 ± 8 | 54 ± 9 |
| Metastatic efficiency, i.e. number of lung colonies per $1 \times 10^6$ viable A-07 cells or per $1.0 \times 10^5$ viable D-12 cells | | | | | | | | | | |
| 0 | 12 ± 4 | 10 ± 3 | 13 ± 3 | 14 ± 3 | 11 ± 4 | 9 ± 2 | 20 ± 4 | 26 ± 6 | 32 ± 7 | 35 ± 6 |
| 4 | 13 ± 3 | 14 ± 4 | 15 ± 3 | 10 ± 5 | 14 ± 4 | 9 ± 1 | 16 ± 3 | 18 ± 4 | 22 ± 5 | 22 ± 5 |
| 24 | 12 ± 4 | 13 ± 4 | 10 ± 5 | 11 ± 5 | 15 ± 5 | 10 ± 3 | 9 ± 3 | 12 ± 2 | 8 ± 3 | 10 ± 3 |

[a]Mean ± s.e.m. of 6–10 experiments, each involving 10 mice, i.e. the value derived from each experiment was the mean number of colonies of 10 pairs of lungs.
[b]Mean ± s.e.m. of 6–10 experiments, each involving 4 culture flasks, i.e. the value derived from each experiment was the mean number of colonies of 4 flasks.

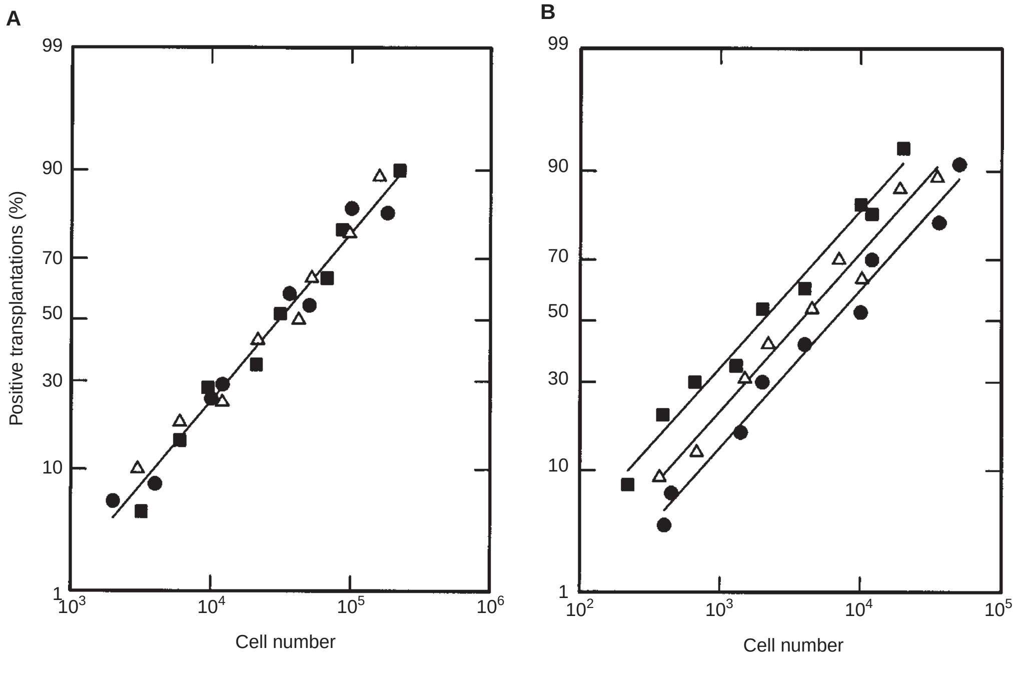

**Figure 3** A-07 (**A**) and D-12 (**B**) cells were exposed to hypoxia in vitro, reoxygenated and inoculated intradermally in female BALB/c-*nu/nu* mice for tumour formation. The mice were examined for up to 120 days after the inoculation. The plots show percentage of positive transplantations vs number of cells per inoculum. The cells were harvested from aerobic control cultures (●) or cultures exposed to hypoxia for 24 h (■, Δ). The hypoxia-treated cells were inoculated immediately (< 1 h) after the reoxygenation (■) or 4 h after the reoxygenation (Δ). Points are based on 20–30 inoculations. Probit regression lines were fitted to the data

Hypoxia treatment also increased transiently the angiogenic potential of the D-12 cells (Figure 4). The magnitude of the increase was independent of the number of cells per inoculum. The kinetics were similar to the kinetics of the transient increases in metastatic efficiency and transplantability. Thus, after a hypoxia treatment of 24 h, the angiogenic potential was significantly higher for cell cultures assayed immediately after the reoxygenation than for cell cultures assayed 4 h after the reoxygenation ($P < 0.01$), which in turn showed a significantly higher angiogenic potential than aerobic control cultures ($P < 0.01$). The hypoxia-induced increase in angiogenic potential was eliminated 24 h after the reoxygenation; the angiogenic potential of cell cultures assayed 24 h after the reoxygenation did not differ significantly from that of aerobic control cultures ($P > 0.05$; data not shown). In contrast,

*© 1999 Cancer Research Campaign* 

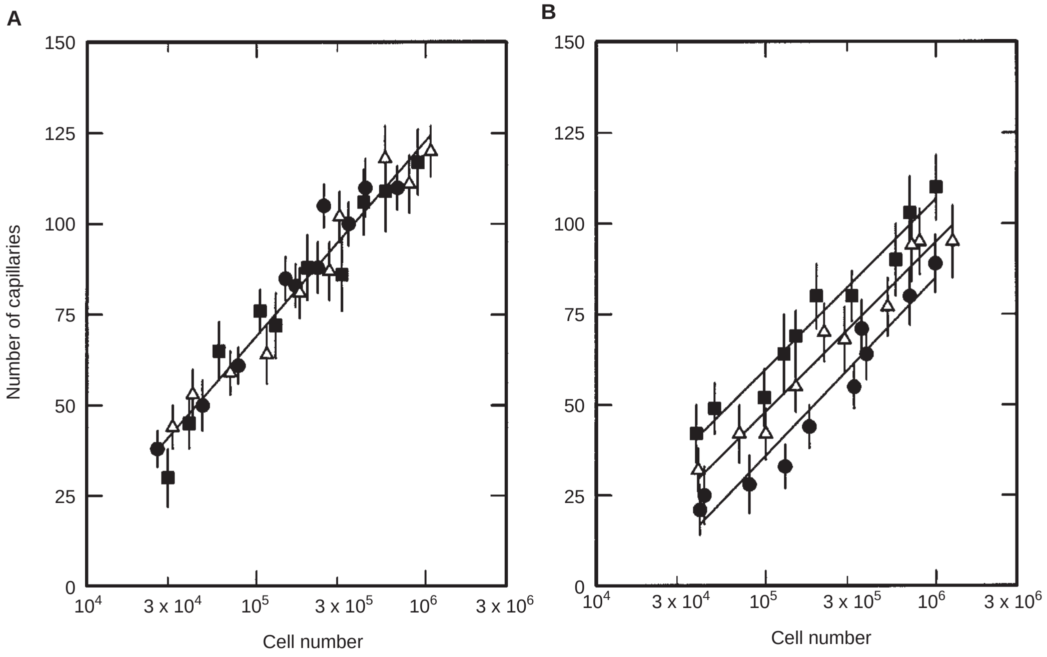

**Figure 4** A-07 (**A**) and D-12 (**B**) cells were exposed to hypoxia in vitro, reoxygenated and inoculated intradermally in female BALB/c-*nu/nu* mice for angiogenesis evocation and tumour formation. Angiogenesis was quantified by counting the capillaries in the dermis oriented towards the tumours at day 5 (A-07) or day 7 (D-12) after the inoculation. The plots show number of tumour-oriented capillaries vs number of cells per inoculum. The cells were harvested from aerobic control cultures (●) or cultures exposed to hypoxia for 24 h (■, Δ). The hypoxia-treated cells were inoculated immediately (< 1 h) after the reoxygenation (■) or 4 h after the reoxygenation (Δ). Mean values (points) and s.e.m. (bars) of 12 tumours. Regression lines were fitted to the data

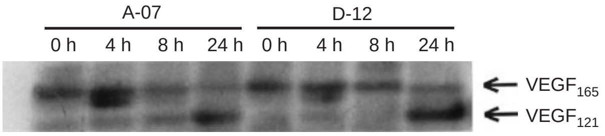

**Figure 5** Western blots representing the intracellular VEGF concentration in A-07 and D-12 cells grown in monolayer culture under hypoxic conditions for 0 (aerobic control cultures), 4, 8 or 24 h. Proteins from $5.0 \times 10^5$ cells were loaded into each lane. Three bands are seen, possibly corresponding to glycosylated $VEGF_{165}$, nonglycosylated $VEGF_{165}$ and $VEGF_{121}$

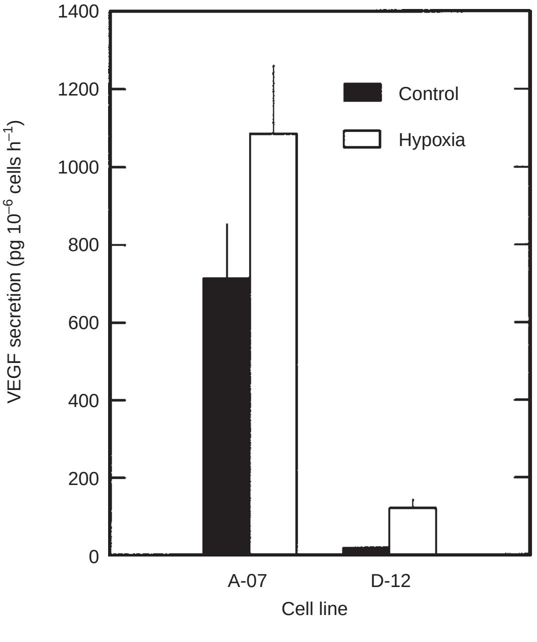

**Figure 6** Rate of VEGF secretion in A-07 and D-12 cells grown in monolayer culture under aerobic or hypoxic conditions. Mean values (columns) and s.e.m. (bars) of 3–7 experiments the angiogenic potential of the A-07 cells was not changed by hypoxia treatment (Figure 4); cell cultures exposed to hypoxia for 24 h and assayed immediately after or 4 h after the reoxygenation showed an angiogenic potential which was not significantly different from that of aerobic control cultures ($P > 0.05$). There was a strong correlation between tumour weight and number of tumour-oriented capillaries for each cell line ($P < 0.0001$; data not shown).

Western blot analysis showed that the concentration of intracellular VEGF protein was similar in A-07 and D-12 cells (Figure 5). Three bands were detected, possibly corresponding to glycosylated $VEGF_{165}$, non-glycosylated $VEGF_{165}$ and $VEGF_{121}$. If so, non-glycosylated $VEGF_{165}$ and $VEGF_{121}$ were up-regulated in cell cultures exposed to hypoxia for about 4 h and 24 h, respectively. In contrast, the rate of VEGF secretion differed between the cell lines (Figure 6). The A-07 cells showed a higher secretion rate than the D-12 cells, both under aerobic ($P < 0.005$) and hypoxic ($P < 0.01$) conditions. The secretion rate was higher for hypoxic cultures than for aerobic cultures by factors of approximately 1.5 (A-07; $P < 0.05$) and 7 (D-12; $P < 0.001$). The absolute increase in VEGF secretion rate was higher for the A-07 cells than for the D-12 cells ($P < 0.05$; Figure 6). Consequently, the D-12 cells, which showed significant hypoxia-induced increases in

*© 1999 Cancer Research Campaign*

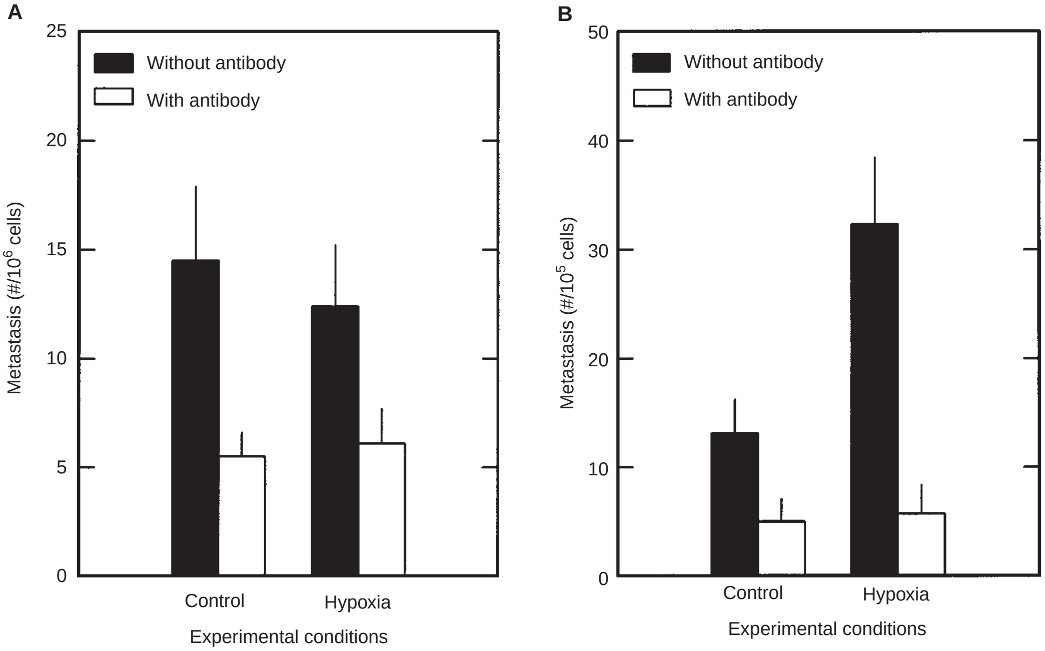

**Figure 7** A-07 (**A**) and D-12 (**B**) cells harvested from aerobic control cultures or cultures exposed to hypoxia in vitro for 24 h were inoculated intravenously in female BALB/c-*nu/nu* mice for formation of lung colonies. The hypoxia-treated cells were inoculated immediately (< 1 h) after the reoxygenation. The lung colonies were scored 5 weeks after the inoculation. The plots show number of lung colonies per $1.0 \times 10^6$ viable A-07 cells or per $1.0 \times 10^5$ viable D-12 cells. Filled columns refer to cells inoculated in untreated mice and open columns to cells inoculated in mice treated with neutralizing antibody against VEGF. Mean values (columns) and s.e.m. (bars) of ten mice

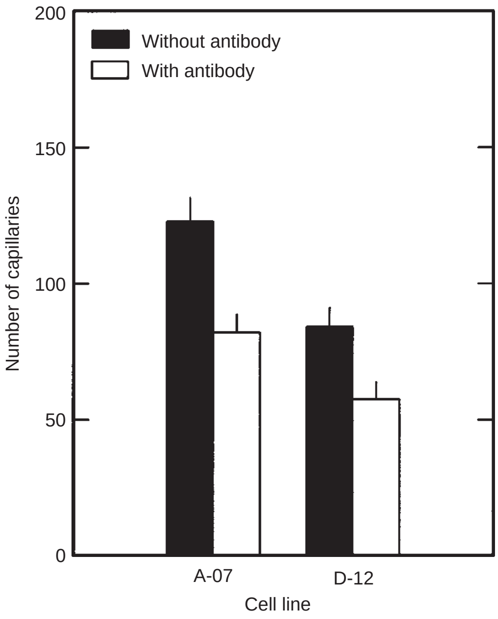

**Figure 8** A-07 and D-12 cells harvested from aerobic cell cultures were inoculated intradermally in female BALB/c-*nu/nu* mice for angiogenesis evocation and tumour formation. Angiogenesis was quantified by counting the capillaries in the dermis oriented towards the tumours at day 5 (A-07) or day 7 (D-12) after the inoculation. The plot shows number of tumour-oriented capillaries after inoculation of $1.0 \times 10^6$ cells. Filled columns refer to cells inoculated in untreated mice and open columns to cells inoculated in mice treated with neutralizing antibody against VEGF. Mean values (columns) and s.e.m. (bars) of 20 tumours metastatic efficiency, transplantability and angiogenic potential, had a lower VEGF secretion rate under aerobic conditions and a lower hypoxia-induced increase in the VEGF secretion rate than the A-07 cells, which did not show significant hypoxia-induced increases in metastatic efficiency, transplantability and angiogenic potential.

Treatment of host mice with neutralizing antibody against VEGF resulted in a decrease in the metastatic efficiency of the D-12 cells (Figure 7). The metastatic efficiency in untreated mice was higher than that in antibody-treated mice by a factor of 2–3 for aerobic control cultures ($P < 0.05$) and by a factor of 5–6 for cell cultures exposed to hypoxia for 24 h and assayed immediately after the reoxygenation ($P < 0.001$). Hypoxia-treated cultures showed a higher metastatic efficiency than aerobic control cultures in untreated mice ($P < 0.05$), consistent with the data in Figure 1. In contrast, the metastatic efficiency of hypoxia-treated cultures was not significantly different from that of aerobic control cultures in antibody-treated mice ($P > 0.05$). Also the A-07 cells showed reduced metastatic efficiency in mice treated with neutralizing antibody against VEGF (Figure 7). The metastatic efficiency in untreated mice was higher than that in antibody-treated mice by a factor of 2–3 for both aerobic control cultures ($P < 0.05$) and cultures assayed immediately after a hypoxia treatment of 24 h ($P < 0.05$). Aerobic control cultures and hypoxia-treated cultures showed metastatic efficiencies that were not significantly different, either in untreated mice ($P > 0.05$) or in antibody-treated mice ($P > 0.05$).

Treatment of host mice with neutralizing antibody against VEGF also resulted in a decrease in the angiogenic potential of the A-07 and D-12 cells (Figure 8). The angiogenic potential of aerobic cell cultures was higher in untreated mice than in antibody-treated mice by a factor of approximately 1.5 for both A-07 ($P < 0.001$) and D-12 ($P < 0.01$).

© 1999 Cancer Research Campaign 

### DISCUSSION

Experimental studies of relationships between tumour metastasis, angiogenesis and concentrations of specific angiogenic factors require the use of adequate tumour models. Two human melanoma lines giving rise to pulmonary metastases in athymic nude mice were used as models in the study reported here. These lines are useful models for studies of molecular mechanisms involved in hypoxia-induced changes in metastatic propensity for several reasons. Thus, both cell lines form distinct, well-defined and hence easily scorable lung colonies following intravenous cell inoculation, and there is a linear relationship between the number of lung colonies and the number of inoculated cells in both lines. Moreover, the difference in metastatic efficiency between the lines is not a result of a difference in intrinsic metastatic propensity, but is rather a result of different host immune reactivities against the lines (Rofstad, 1995).

The study reported here has shown that the metastatic efficiency of human tumour cells can be enhanced by hypoxia. Thus, D-12 cell cultures exposed to hypoxia in vitro, reoxygenated and inoculated i.v. in athymic nude mice showed increased lung colonization efficiency relative to aerobic control cultures. However, hypoxia does not necessarily increase the metastatic efficiency of the cells of all tumours; the lung colonization efficiency of the A-07 cells was not enhanced by hypoxia treatment in vitro. Young et al (1988) have reported previously that exposure to hypoxia in vitro can increase the lung colonization efficiency of murine tumour cells (KHT-C2-LP1 fibrosarcoma and B16F10-A1 melanoma) and attributed the effect to hypoxia-induced DNA-overreplication and unspecific gene amplification. No overall temporal correlation between the increase in lung colonization efficiency following exposure to hypoxia and hypoxia-induced changes in the *m*-RNA levels for cathepsin B, cathepsin L, nm23, TIMP-1, osteopontin or VEGF was found for these murine tumour cells (Jang and Hill, 1997).

The mechanism of the hypoxia-induced increase in metastatic efficiency in the D-12 cells was probably fundamentally different from that in the murine tumour cells studied by Young et al (1988). Firstly, the kinetics as well as the magnitude of the effect differed significantly between the D-12 and the murine tumour cells. When the murine tumour cells were exposed to hypoxia for 18–24 h, the metastatic efficiency was reduced slightly immediately after the reoxygenation, increased with the reoxygenation time, reached a maximum at about 18 h after the reoxygenation and then decayed with the reoxygenation time. In contrast, the metastatic efficiency of the D-12 cells was highest immediately after the reoxygenation, decreased with the reoxygenation time and was similar to that of aerobic control cultures at about 24 h after the reoxygenation. At maximum, the metastatic efficiency was enhanced by a factor of 6–14 in the murine tumour cells and by a factor of only 3–4 in the D-12 cells. Secondly, in contrast to the murine tumour cells, the D-12 cells did probably not show unspecific gene amplification following exposure to hypoxia, as DNA-overreplication similar to that observed by Young et al (1988) in the studies of the murine KHT-C2-LP1 fibrosarcoma and B16F10-A1 melanoma cells could not be detected when D-12 cells were subjected to flow cytometric analyses.

The D-12 cells increased slightly in volume during prolonged (> 8 h) exposure to hypoxia. Increased cell volume might promote the formation of metastases by facilitating the arrest of tumour cells in the microcirculation. Prolonged hypoxia treatment also resulted in inactivation of a significant fraction of the D-12 cells. Since cell inactivation by hypoxia can be cell cycle-dependent (Spiro et al, 1984), the cell cycle distribution of the non-inactivated D-12 cells might have differed from that of the aerobic control cells. The lung colonization ability of cells can depend on their position in the cell cycle (Suzuki et al, 1977). However, it is unlikely that the hypoxia-induced increase in metastatic efficiency in the D-12 cells can be attributed to hypoxia-induced alterations in cell volume or cell cycle distribution. The hypoxia-induced alterations in cell volume and PE were similar in the D-12 and the A-07 cells, and the A-07 cells did not show increased metastatic efficiency following exposure to hypoxia. Moreover, the metastatic efficiency of the D-12 cells was enhanced by a factor of 2–3 immediately after a hypoxia treatment of 4–8 h, and a hypoxia treatment of 4–8 h did not cause significant alterations in cell volume or PE.

The hypoxia-induced increase in metastatic efficiency in the D-12 cells was rather a result of a hypoxia-induced increase in angiogenic potential. Thus, the D-12 cells, which showed hypoxia-induced metastasis, evoked increased angiogenesis following exposure to hypoxia, whereas the A-07 cells, which did not show hypoxia-induced metastasis, did not. Moreover, tumour transplantability, which also is angiogenesis-dependent, was enhanced following hypoxia treatment in the D-12 cells but not in the A-07 cells. Finally, the kinetics of the hypoxia-induced increases in metastasis and transplantability were similar to that of the hypoxia-induced increase in angiogenic potential.

VEGF was probably involved in the hypoxia-induced angiogenesis causing the enhanced metastatic efficiency of hypoxia-treated D-12 cells. Thus, non-glycosylated $VEGF_{165}$ and $VEGF_{121}$ were clearly up-regulated in cell cultures exposed to hypoxia for about 4 h and 24 h respectively. The rate of VEGF secretion in vitro was higher under hypoxic conditions than under aerobic conditions by a factor of approximately 7. Moreover, the metastatic efficiency and the angiogenic potential of the D-12 cells were reduced significantly in mice treated with neutralizing antibody against VEGF. Hypoxia-treated and aerobic control cultures showed similar metastatic efficiencies in antibody-treated mice. However, the present study does of course not exclude the possibility that angiogenic factors other than VEGF also were involved in the hypoxia-induced increases in metastatic efficiency and angiogenic potential in the D-12 cells.

The neovascularization and expansion of micrometastases do not occur until several days after their establishment. Northern blot analyses have shown that hypoxia-induced VEGF up-regulation in vitro decreases with the time after reoxygenation with a half-life of a few hours (Shweiki et al, 1992; Hlatky et al, 1994), consistent with the observation that a reoxygenation time of 24 h, but not a reoxygenation time of 4 h, was sufficient to eliminate the hypoxia-induced increase in metastatic efficiency in the D-12 cells. Consequently, the later phases of the angiogenic process were probably not influenced significantly by exposing the D-12 cells to hypoxia. It is predominantly more likely that VEGF contributed to the hypoxia-induced increase in the metastatic efficiency of the D-12 cells by potentiating the initial phase of angiogenesis, i.e. by stimulating the expression of endothelial cell proteases and hence the degradation of the basement membrane (Unemori et al, 1992; Mandriota et al, 1995).

Perhaps more importantly, VEGF may have contributed significantly to the increased metastatic efficiency of hypoxia-treated D-12 cells by a mechanism which is only partly related to the

© 1999 Cancer Research Campaign process of angiogenesis. VEGF is known also as vascular permeability factor based on its ability to induce vascular leakage in several organs, including skin and peritoneal wall (Senger et al, 1983; Dvorak, 1986; Nagy et al, 1995). VEGF increases microvascular permeability through activation of vesicular-vacuolar organelles in endothelial cells (Feng et al, 1996) and through the induction of inter-endothelial cell gaps and endothelial fenestration (Roberts and Palade, 1995). The vascular permeabilizing effect occurs rapidly, becoming evident within several minutes after VEGF exposure (Dvorak et al, 1979; Senger et al, 1983; Nagy et al, 1995). VEGF may therefore have increased the metastatic efficiency of hypoxia-treated D-12 cells by facilitating cell extravasation into the lungs. Moreover, increased microvascular permeability is probably also a crucial step in tumour angiogenesis, as Dvorak (1986) has presented evidence that a major function of VEGF in the angiogenic process is the induction of plasma protein leakage, an effect which results in the formation of an extravascular fibrin gel serving as a substrate for endothelial and tumour cell growth. Consequently, the vascular permeabilization effect of VEGF may have contributed to the hypoxia-induced increase in the metastatic efficiency of the D-12 cells not only by assisting cell extravasation, but also by potentiating tumour angiogenesis after the extravasation. Thus, this interpretation of the metastasis data is consistent with the observation that the transplantability of the D-12 cells was increased by exposure to hypoxia.

Also the A-07 cells showed VEGF upregulation under hypoxic conditions in vitro, but these cells, in contrast to the D-12 cells, did not show hypoxia-induced metastasis and angiogenesis. Several observations suggest that the lack of hypoxia-induced metastasis and angiogenesis in the A-07 cells was not due to the VEGF of these cells being inactive in vivo. Thus, the metastatic efficiency and the angiogenic potential of the A-07 cells were reduced significantly in mice treated with neutralizing antibody against VEGF, similar to what was seen for the D-12 cells. Moreover, ten of ten mice have been found to develop ascitic fluid following intraperitoneal (i.p.) inoculation of $1.0 \times 10^6$ A-07 cells, whereas i.p. inoculation of $1.0 \times 10^6$ D-12 cells resulted in the development of ascites in only one of ten mice (unpublished data). Similar studies of human ovarian carcinomas in athymic nude mice have shown that the production of ascites is directly associated with the expression of active VEGF (Yoneda et al, 1998). Finally, the Western blot analyses suggested that the same isoforms of VEGF were expressed by A-07 and D-12 cells, both under aerobic and hypoxic conditions.

The lack of hypoxia-induced metastasis and angiogenesis in the A-07 cells was probably rather a consequence of the high expression of VEGF under aerobic conditions; the aerobic rate of VEGF secretion was higher in the A-07 cells than in the D-12 cells by a factor of approximately 40. It is therefore possible that VEGF was a limiting factor for the development of metastases and the rate of angiogenesis in the D-12 cells but not in the A-07 cells. If so, exposure of the A-07 cells to hypoxia just led to increased secretion of redundant VEGF.

The present study has significant influence on our understanding of the role of VEGF in the angiogenesis of malignant melanoma. If the data presented here are representative for melanomas in man, three important conclusions may be derived. Firstly, melanomas in different patients can differ substantially in VEGF expression. Secondly, VEGF may be a limiting factor for the rate of angiogenesis in low VEGF-expressing melanomas but not in high VEGF-expressing melanomas under normoxic conditions. Thirdly, the VEGF expression in low VEGF-expressing melanomas may be up-regulated by hypoxia to an extent sufficient to increase the rate of angiogenesis. Consequently, the rate of VEGF secretion might be an important determinant of the rate of angiogenesis in malignant melanoma. This suggestion is consistent with the observation that human melanoma xenografts transfected with VEGF can show increased vascular density relative to non-transfected control tumours (Claffey et al, 1996; Pötgens et al, 1996).

Our understanding of the mechanisms governing the development of metastases in malignant melanoma is also influenced significantly by the present study. Melanomas gradually develop aggressive phenotypic traits with time, including invasive growth and metastatic spread (Herlyn, 1990), a property they have in common with several histological types of cancer (Foulds, 1975). This process is termed malignant progression and has been suggested to be a result of genomic instability (Hill, 1990). Evidence has been presented that tumour hypoxia followed by reoxygenation might increase the genomic instability of tumours and hence promote the formation of metastatic cell phenotypes (Hill, 1990; Brown and Giaccia, 1994; Dachs and Stratford, 1996). The study reported here suggests an additional mechanism by which acute hypoxia might promote the development of metastases in melanoma. Melanoma cells intravasating from tumour regions subjected to transient stoppages in microregional blood flow might show up-regulated VEGF expression and hence have elevated capacity to extravasate, induce neovascularization and give rise to macroscopic metastatic growth when trapped in the capillary bed of a distant organ such as the lungs. It should be noted that our data suggest that this mechanism of hypoxia-induced metastasis applies only to melanomas showing low VEGF expression under normoxic conditions and that the increased metastatic efficiency induced during hypoxia is a transient phenomenon; the metastatic efficiency might be significantly elevated at least until 4 h after the reoxygenation, but probably not until 24 h after the reoxygenation.

The mechanism of hypoxia-induced metastasis suggested here is consistent with data from clinical studies involving soft tissue sarcoma, cervix carcinoma and carcinoma of the head and neck showing that primary tumours with low oxygen tensions or high lactate concentrations metastasize more frequently than primary tumours with high oxygen tensions or low lactate concentrations (Schwickert et al, 1995; Brizel et al, 1996; Höckel et al, 1996; Walenta et al, 1997; Sundfør et al, 1998). It is also consistent with observations showing that the probability of metastasis in many tumour types is positively correlated to the vascular density in vascular ‘hot spots’ of the primary tumour, as vascular ‘hot spots’ might result from hypoxia-induced angiogenesis mediated by VEGF (Weidner, 1993).

### ACKNOWLEDGEMENTS

We thank Berit Mathiesen, Kanthi Galappathi, Kristin Nilsen, Heidi Kongshaug and Anne Wahl for skillful technical assistance. Financial support was received from The Norwegian Cancer Society.

### REFERENCES

Asano M, Yukita A, Matsumoto T, Kondo S and Suzuki H (1995) Inhibition of tumor growth and metastasis by an immunoneutralizing monoclonal antibody to human vascular endothelial growth factor/vascular permeability factor$_{121}$. *Cancer Res* **55**: 5296–5301

Bochner BH, Cote RJ, Weidner N, Groshen S, Chen SC, Skinner DG and Nichols PW (1995) Angiogenesis in bladder cancer: relationship between microvessel density and tumor prognosis. *J Natl Cancer Inst* **87**: 1603–1612

Brizel DB, Scully SP, Harrelson JM, Layfield LJ, Bean JM, Prosnitz LR and Dewhirst MW (1996) Tumor oxygenation predicts for the likelihood of distant metastases in human soft tissue sarcoma. *Cancer Res* **56**: 941–943

Brown JM and Giaccia AJ (1994) Tumour hypoxia: the picture has changed in the 1990s. *Int J Radiat Biol* **65**: 95–102

Claffey KP, Brown LF, del Aguila LF, Tognazzi K, Yeo K-T, Manseau EJ and Dvorak HF (1996) Expression of vascular permeability factor/vascular endothelial growth factor by melanoma cells increases tumor growth, angiogenesis, and experimental metastasis. *Cancer Res* **56**: 172–181

Coleman CN (1988) Hypoxia in tumors: a paradigm for the approach to biochemical and physiologic heterogeneity. *J Natl Cancer Inst* **80**: 310–317

Dachs GU and Stratford IJ (1996) The molecular response of mammalian cells to hypoxia and the potential for exploitation in cancer therapy. *Br J Cancer* **74**: s126–s132

Denekamp J and Hobson B (1982) Endothelial cell proliferation in experimental tumours. *Br J Cancer* **46**: 711–720

Dvorak HF (1986) Tumours: wounds that do not heal. Similarity between tumor stroma generation and wound healing. *New Engl J Med* **315**: 1650–1658

Dvorak HF, Orenstein NS, Carvalho AC, Churchill WH, Dvorak AM, Galli SJ, Feder J, Bitzer AM, Rypysc J and Giovinco P (1979) Induction of a fibrin-gel investment: an early event in line 10 hepatocarcinoma growth mediated by tumor-secreted products. *J Immunol* **122**: 166–174

Feng D, Nagy JA, Hipp J, Dvorak HF and Dvorak AM (1996) Vesiculo-vacuolar organelles and the regulation of venule permeability to macromolecules by vascular permeability factor, histamine, and serotonin. *J Exp Med* **183**: 1981–1986

Fidler IJ and Ellis LM (1994) The implications of angiogenesis for the biology and therapy of cancer metastasis. *Cell* **79**: 185–188

Folkman J (1990) What is the evidence that tumors are angiogenesis dependent? *J Natl Cancer Inst* **82**: 4–6

Foulds L (1975) *Neoplastic Development*: Academic Press: New York

Gasparini G, Weidner N, Maluta S, Pozza F, Boracchi P, Mezzetti M, Testolin A and Bevilacqua P (1993) Intratumoral microvessel density and p53 protein: correlation with metastasis in head-and-neck squamous-cell carcinoma. *Int J Cancer* **55**: 739–744

Guidi AJ, Abu-Jawdeh G, Berse B, Jackman RW, Tognazzi K, Dvorak HF and Brown LF (1995) Vascular permeability factor (vascular endothelial growth factor) expression and angiogenesis in cervical neoplasia. *J Natl Cancer Inst* **87**: 1237–1245

Herlyn M (1990) Human melanoma: development and progression. *Cancer Metastasis Rev* **9**: 101–112

Hill RP (1990) Tumor progression: potential role of unstable genomic changes. *Cancer Metastasis Rev* **9**: 137–147

Hlatky L, Tsionou C, Hahnfeldt P and Coleman CN (1994) Mammary fibroblasts may influence breast tumor angiogenesis via hypoxia-induced vascular endothelial growth factor up-regulation and protein expression. *Cancer Res* **54**: 6083–6086

Horsman MR (1995) Nicotinamide and other benzamide analogs as agents for overcoming hypoxic cell radiation resistance in tumours. *Acta Oncol* **34**: 571–587

Höckel M, Schlenger K, Aral B, Mitze M, Schäffer U and Vaupel P (1996) Association between tumor hypoxia and malignant progression in advanced cancer of the uterine cervix. *Cancer Res* **56**: 4509–4515

Jang A and Hill RP (1997) An examination of the effects of hypoxia, acidosis, and glucose starvation on the expression of metastasis-associated genes in murine tumor cells. *Clin Exp Metastasis* **15**: 469–483

Kim KJ, Li B, Winer J, Armanini M, Gillett N, Phillips HS and Ferrara N (1993) Inhibition of vascular endothelial growth factor-induced angiogenesis suppresses tumour growth in vivo. *Nature* **362**: 841–844

Kreisle RA and Ershler WB (1988) Investigation of tumor angiogenesis in an id mouse model: role of host–tumor interactions. *J Natl Cancer Inst* **80**: 849–854

Laemmli UK (1970) Clevage of structural proteins during the assembly of the head of bacterio-phage T4. *Nature* **277**: 680–685

Macchiarini P, Fontanini G, Hardin MJ, Squartini F and Angeletti CA (1992) Relation of neovasculature to metastasis of non-small-cell lung cancer. *Lancet* **340**: 145–146

Mandriota SJ, Seghezzi G, Vassalli JD, Ferrara N, Wasi S, Mazzieri R, Mignatti P and Pepper MS (1995) Vascular endothelial growth factor increases urokinase receptor expression in vascular endothelial cells. *J Biol Chem* **270**: 9707–9716

Mattern J, Koomägi R and Volm M (1996) Association of vascular endothelial growth factor expression with intratumoral microvessel density and tumour cell proliferation in human epidermoid lung carcinoma. *Br J Cancer* **73**: 931–934

Melnyk O, Shuman MA and Kim KJ (1996) Vascular endothelial growth factor promotes tumor dissemination by a mechanism distinct from its effect on primary tumor growth. *Cancer Res* **56**: 921–924

Nagy JA, Masse EM, Herzberg KT, Meyers MS, Yeo KT, Yeo TK, Sioussat TM and Dvorak HF (1995) Pathogenesis of ascites tumor growth. Vascular permeability factor, vascular hyperpermeability and ascites fluid accumulation. *Cancer Res* **55**: 360–368

Pötgens AJG, van Altena MC, Lubsen NH, Ruiter DJ and de Waal RMW (1996) Analysis of the tumor vasculature and metastatic behavior of xenografts of human melanoma cell lines transfected with vascular permeability factor. *Am J Pathol* **148**: 1203–1217

Roberts WG and Palade GE (1995) Increased microvascular permeability and endothelial fenestration induced by vascular endothelial growth factor. *J Cell Sci* **108**: 2369–2379

Rofstad EK (1992) Retention of cellular radiation sensitivity in cell and xenograft lines established from human melanoma surgical specimens. *Cancer Res* **52**: 1764–1769

Rofstad EK (1994) Orthotopic human melanoma xenograft model systems for studies of tumour angiogenesis, pathophysiology, treatment sensitivity and metastatic pattern. *Br J Cancer* **70**: 804–812

Rofstad EK (1995) Metastatic behavior of human tumors in congenitally athymic nude mice: intrinsic properties of the tumor cells and host immune reactivity. *Int J Cancer* **63**: 744–749

Saleh M, Stacker SA and Wilks AF (1996) Inhibition of growth of C6 glioma cells in vivo by expression of antisense vascular endothelial growth factor sequence. *Cancer Res* **56**: 393–401

Sanna K and Rofstad EK (1994) Hypoxia-induced resistance to doxorubicin and methotrexate in human melanoma cell lines in vitro. *Int J Cancer* **58**: 258–262

Schwickert G, Walenta S, Sundfør K, Rofstad EK and Mueller-Klieser W (1995) Correlation of high lactate levels in human cervical cancer with incidence of metastasis. *Cancer Res* **55**: 4757–4759

Senger DR, Galli SJ, Dvorak AM, Perruzzi CA, Harvey VS and Dvorak HF (1983) Tumor cells secrete a vascular permeability factor that promotes accumulation of ascites fluid. *Science* **219**: 983–985

Shweiki D, Itin A, Soffer D and Keshet E (1992) Vascular endothelial growth factor induced by hypoxia may mediate hypoxia-initiated angiogenesis. *Nature* **359**: 843–845

Spiro IJ, Rice GC, Durand RE, Stickler R and Ling CC (1984) Cell killing, radiosensitization, and cell cycle redistribution induced by chronic hypoxia. *Int J Radiat Oncol Biol Phys* **10**: 1275–1280

Sundfør K, Lyng H and Rofstad EK (1998) Tumour hypoxia and vascular density as predictors of metastasis in squamous cell carcinoma of the uterine cervix. *Br J Cancer* **78**: 822–827

Suzuki N, Frapart M, Grdina JD, Meistrich ML and Withers R (1977) Cell cycle dependency of metastatic lung colony formation. *Cancer Res* **37**: 3690–3693

Takahashi Y, Kitadai Y, Bucana CD, Cleary KR and Ellis LM (1995) Expression of vascular endothelial growth factor and its receptor, KDR, correlates with vascularity, metastasis, and proliferation of human colon cancer. *Cancer Res* **55**: 3964–3968

Toi M, Inada K, Hoshina S, Suzuki H, Kondo S and Tominaga T (1995) Vascular endothelial growth factor and platelet-derived endothelial cell growth factor are frequently co-expressed in highly vascularized human breast cancer. *Clin Cancer Res* **1**: 961–964

Towbin H, Staehelin T and Gordon J (1979) Electrophoretic transfer of proteins from poly-acrylamide gels to nitrocellulose sheets: procedure and some applications. *Proc Natl Acad Sci USA* **76**: 4350–4354

Unemori EN, Ferrara N, Bauer EA and Amento EP (1992) Vascular endothelial growth factor induces interstitial collagenase expression in human endothelial cells. *J Cell Physiol* **153**: 557–562

Vaupel P, Kallinowski F and Okunieff P (1989) Blood flow, oxygen and nutrient supply, and metabolic microenvironment of human tumors: a review. *Cancer Res* **49**: 6449–6465

Walenta S, Salameh A, Lyng H, Evensen JF, Mitze M, Rofstad EK and Mueller-Klieser W (1997) Correlation of high lactate levels in head and neck tumors with incidence of metastasis. *Am J Pathol* **150**: 409–415

Weidner N (1993) Tumor angiogenesis: review of current applications in tumor prognostication. *Semin Diagn Pathol* **10**: 302–313

Weidner N, Semple JP, Welch WR and Folkman J (1991) Tumor angiogenesis and metastasis – correlation in invasive breast carcinoma. *N Engl J Med* **324**: 1–8

*© 1999 Cancer Research Campaign*

Yoneda J, Kuniyasu H, Crispens MA, Price JE, Bucana CD and Fidler IJ (1998) Expression of angiogenesis-related genes and progression of human ovarian carcinomas in nude mice. *J Natl Cancer Inst* **90**: 447–454

Young SD, Marshall RS and Hill RP (1988) Hypoxia induces DNA overreplication and enhances metastatic potential of murine tumor cells. *Proc Natl Acad Sci USA* **85**: 9533–9537

Zagzag D (1995) Angiogenic growth factors in neural embryogenesis and neoplasia. *Am J Pathol* **146**: 293–309

Zhang H-T, Craft P, Scott PAE, Ziche M, Weich HA, Harris AL and Bicknell R (1995) Enhancement of tumor growth and vascular density by transfection of vascular endothelial cell growth factor into MCF-7 human breast carcinoma cells. *J Natl Cancer Inst* **87**: 213–219

© 1999 Cancer Research Campaign
